# Humoral Immune Response after the Third SARS-CoV-2 mRNA Vaccination in CD20 Depleted People with Multiple Sclerosis

**DOI:** 10.3390/vaccines9121470

**Published:** 2021-12-11

**Authors:** Lutz Achtnichts, Barbara Jakopp, Michael Oberle, Krassen Nedeltchev, Christoph Andreas Fux, Johann Sellner, Oliver Findling

**Affiliations:** 1 Department of Neurology, Aarau Cantonal Hospital, 5000 Aarau, Switzerland; Lutz.Achtnichts@ksa.ch (L.A.); Krassen.Nedeltchev@ksa.ch (K.N.); 2 Department of Infectious Diseases and Hospital Infection Prevention, Aarau Cantonal Hospital, 5000 Aarau, Switzerland; Barbara.jakopp@ksa.ch (B.J.); Christoph.Fux@ksa.ch (C.A.F.); 3 Institute for Laboratory Medicine, Aarau Cantonal Hospital, 5000 Aarau, Switzerland; Michael.Oberle@ksa.ch; 4 Department of Neurology, University of Bern, 3012 Bern, Switzerland; 5 Department of Neurology, Landesklinikum Mistelbach-Gänserndorf, 2130 Mistelbach, Austria; Johann.Sellner@mistelbach.lknoe.at; 6 MS Center and Research Center for Clinical Neuroimmunology and Neuroscience Basel (RC2NB), Head, Spine and Neuromedicine, Clinical Research and Biomedicine and Biomedical Engineering, University Hospital and University of Basel, 4031 Basel, Switzerland

**Keywords:** SARS-CoV-2, B cell depletion, humoral immune response, vaccination, COVID-19

## Abstract

CD20 depletion is a risk factor for unfavorable outcomes of COVID-19 in people with MS (pwMS). Evidence suggests that protective IgG response to mRNA-based vaccines in B cell-depleted individuals is limited. We studied the seroconversion after the third mRNA SARS-CoV-2 vaccine in B cell-depleted pwMS with limited or no IgG response after the standard immunization. Sixteen pwMS treated with ocrelizumab or rituximab received a third homologous SARS-CoV-2 mRNA vaccine, either the Moderna mRNA-1273 or Pfizer-BioNTech’s BNT162b2 vaccine. We quantified the response of IgG antibodies against the spike receptor-binding domain of SARS-CoV-2 four weeks later. An antibody titer of 100 AU/mL or more was considered clinically relevant. The median time between the last infusion of the anti-CD20 treatment and the third vaccination was 22.9 weeks (range 15.1–31.3). After the third vaccination, one out of 16 patients showed an IgG titer deemed clinically relevant. Only the seroconverted patient had measurable B-cell counts at the time of the third vaccination. The development of a humoral immune response remains rare in pwMS on anti-CD20 therapy, even after third dose of the homologous SARS-CoV-2 mRNA vaccine. It remains to be determined whether T-cell responses can compensate for the lack of seroconversion and provide sufficient protection against CoV-2 infections.

## 1. Introduction

Coronavirus infectious disease 2019 (COVID-19), a multi-organ disease caused by severe acute respiratory syndrome coronavirus 2 (SARS-CoV-2), continues to take its toll on health, medical care, and economy [1]. Strategies to end the pandemic include the implementation of vaccination campaigns and measures to contain the disease [2,3]. The highly transmissible nature of the Delta variant, ongoing vaccine hesitancy, and inconsistent public-health measures represent challenges [4,5]. The mRNA-based BNT162b2 (Pfizer–BioNTech, BioNTech Manufacturing GmbH, 55131 Mainz, Germany) and mRNA-1273 (Moderna Biotech Spain, S.L., 28010 Madrid, Spain) vaccines proved efficacious in phase 3 trials and were the only licensed vaccines against SARS-CoV-2 in Switzerland until October 2021 [6,7]. They exert their effect via nucleoside-modified mRNA encoding for the prefusion spike glycoprotein of SARS-CoV-2. However, there is increasing evidence that people with multiple sclerosis (pwMS) treated with high-efficacy disease-modifying drugs (DMD), particularly those targeting B cells, have an impaired humoral response to vaccination with mRNA vaccines [8,9,10]. This observation is of concern, as pwMS are also at increased risk for acquiring infections. Furthermore, bacterial and viral infections, e.g., upper respiratory tract infections, can trigger new or worsening MS symptoms in the form of relapses or pseudo-relapses [11]. The susceptibility for infectious disease increases with immunosuppressive medication approved for the treatment of MS [12,13]. Moreover, CD20 depleting monoclonal antibodies rituximab (RTX) and ocrelizumab (OCR) were repeatedly shown to be associated with severe COVID-19 infection and unfavorable outcome [14,15,16,17,18,19,20]. The Swiss MS Society recommended, in August 2021, administration of a third vaccine dose in MS patients treated with B cell-targeted disease-modifying therapies and an insufficient humoral immune response after two vaccine doses [21].

Here, we report the seroconversion rates of pwMS treated with the CD20-depleting monoclonal antibodies after a third dose of an mRNA vaccine in pwMS who had not developed protective IgG responses to two prior immunization courses.

## 2. Materials and Methods

We conducted an observational cross-sectional study of all pwMS treated with OCR or RTX at the MS outpatient clinic of the Cantonal Hospital Aarau, Switzerland. The ethical committee for Northwest and Central Switzerland approved the study (permit number 2016–02233), and all patients gave written consent.

### 2.1. Data Collection

We searched patient records of all pwMS treated with OCR or RTX between August and October 2021. The data were analyzed using descriptive statistics. We used GraphPad Prism version 9 for Windows (GraphPad Software, La Jolla, CA, USA, www.graphpad.com, accessed on 29 October 2021) for the statistical analysis.

### 2.2. Antibody Detection

Antibody titers were measured using Abbott’s SARS-CoV-2 IgG II Quant Assay (Abbott Ireland, Diagnostics Division, Ireland). The SARS-CoV-2 IgG II Quant Assay is a chemiluminescent microparticle immunoassay for the qualitative and quantitative detection of IgG anti-spike antibodies binding to the receptor-binding domain (RBD) of the S1 subunit of the spike protein of SARS-CoV-2 in human serum and plasma on the Alinity-i-System by Abbott. The assay is used for both the detection of past infection and the quantification of vaccine responses. The assay was performed following the manufacturer’s instructions.

### 2.3. Fluorescence Activated Cell Sorting (FACS)

Flowcytometric measurements were carried out on a BD FACSLyric (Becton Dickinson, BD Life Sciences, San Jose, CA, 95131, USA) by measuring 150,000 events of stained sample. Staining was performed by incubating 100 μL EDTA-anticoagulated peripheral blood with anti-CD19 PE (Becton Dickinson, Clone SjJ25C1, BD Life Sciences, San Jose, CA, 95131, USA), anti-CD20 APC (Becton Dickinson, Clone L27, BD Life Sciences, San Jose, CA, 95131, USA), anti-CD45 PerCP (Becton Dickinson, Clone 2D1, BD Life Sciences, San Jose, CA, 95131, USA), and anti-CD38 FITC (Becton Dickinson, Clone HB-7, BD Life Sciences, San Jose, CA, 95131, USA) for 10 min at room temperature in the dark. Cells were then lysed for 10 min with 1× BDlysing solution, washed once with BDFACS Flow Solution, and resuspended therein for immediate acquisition. FCS files were analyzed with FACSSuite. B-cells were identified as CD19 expressing lymphocytes, excluding Plasmablasts/Plasmacells, based on their CD38 expression. CD19 positive B-cells were evaluated for CD20 co-expression.

### 2.4. Clinical Procedure

Following the recommendation of the Swiss MS Society, all pwMS treated with OCR or RTX were approached to assess the development of anti-spike IgG after a full course of SARS-CoV-2 vaccination [21]. Blood was drawn at four weeks, at the earliest, after the second dose to quantify the IgG response against the spike protein. We offered a third vaccination if the antibody response was insufficient and measured the titer again four weeks later. Despite an official cut-off value of 50 AU/mL, we considered, according to recommendations, an antibody titer below 100 AU/mL as insufficient [22]. Following the national recommendations, the third vaccination was performed with the same vaccine as the first and second vaccinations [23]. The initial dosing regimen of OCR is 300 mg IV 2 weeks apart, repeated by 600 mg IV every 6 months. The dosing regimen of RTX is 1000 mg IV 2 weeks apart, repeated with 1000 mg every 6 months.

## 3. Results

We identified 51 pwMS treated with OCR or RTX who underwent antibody detection at least four weeks after a second dose of the mRNA-1273 or BNT162b2 vaccines and had no history of COVID-19 infection. Full datasets about disease history and vaccination were available in 49 patients. Fourteen patients (29%) were vaccinated with mRNA-1273, whereas 35 (71%) received BNT162b2. The median time since treatment initiation with OCR or RTX was 2.2 years (range 0.25 to 4.1). The median time between OCR or RTX infusion and the first vaccination was 15.9 weeks (range 5.6 to 41.2). The left part of Table 1 shows the demographical data of the entire cohort and lymphocyte counts at the time point of antibody testing.

Overall, 2/49 (4%) patients had a positive antibody response after two vaccination courses. Patient 1 was a 53.7-year-old man with RRMS (Expanded disability status scale (EDSS): 3.0, disease duration 17.7 years, and OCR treatment for one year at the time of first vaccination). He was vaccinated with BNT162b2 24 weeks after his last OCR infusion and had an antibody titer of 147 AU/mL 27 weeks after the second vaccination. His lymphocyte count was 16 (normal value 200–40). Patient 2 was a 39-year-old woman with RRMS (EDSS: 1.0, disease duration 20.7 years, and OCR treatment for five months at the time of first vaccination) vaccinated with the mRNA-1273 (Moderna) 19 weeks after her last OCR infusion. She had an antibody titer of 366 AU/mL 24 weeks after the second vaccination. Her lymphocyte count was 35 (normal value 20–40)

**Figure 1:**
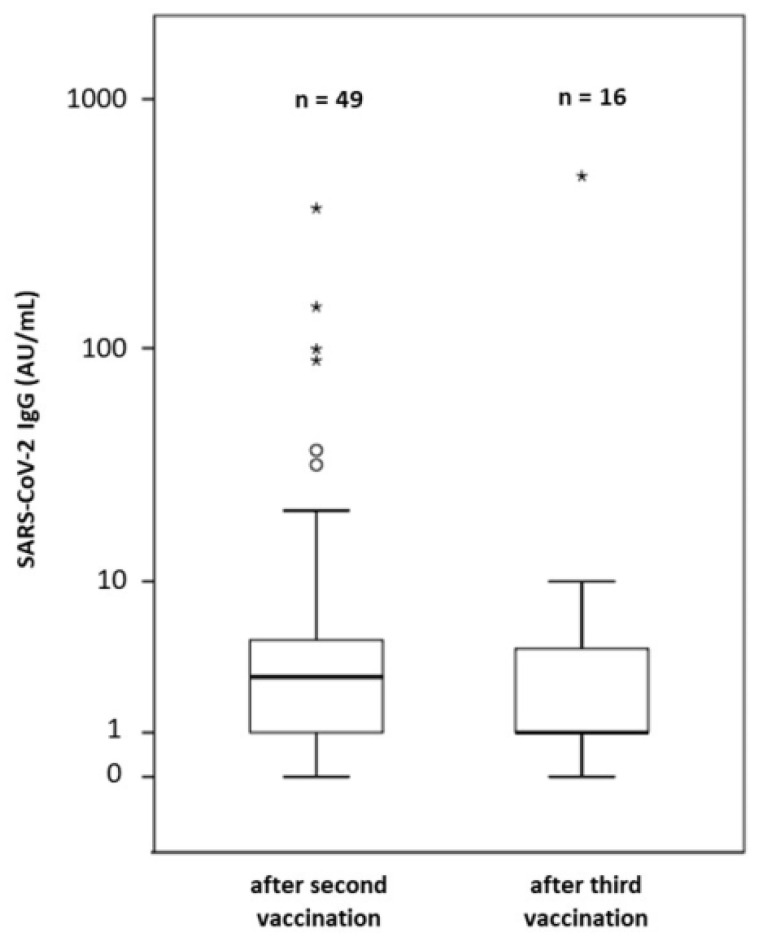
Logarithmic plots of antibody titers after the second and third vaccination against SARS-CoV-2. * represent antibody titers considered clinically relevant.

Sixteen of 47 (34%) patients opted for a third dose. This was performed with the same vaccine as the initial doses at least four months after the last OCR or RTX. Three patients (19%) received the mRNA-1273 and 13 patients (81%) the BNT162b2 vaccine. The median time between the last infusion of OCR or RTX and the third vaccination was 22.9 weeks (range 15.1 to 31.3). The median time between the second and the third vaccination was 14.9 weeks (range 6.6 to 30.1). The right-hand side of Table 1 shows the demographical data of all patients and lymphocyte counts at the time point of antibody testing. The median lymphocyte count at the time point of third vaccination was 1.4 G/L (range 0.9–2.8, normal value 0.8–4 G/L). None of the patients had lymphopenia. Data from Fluorescence Activated Cell Sorting (FACS) to assess B lymphocyte counts at the time point of third vaccination were available in seven patients. All but one patient had effective B cell depletion and had no detectable peripheral blood B cells. This patient was the only one out of 16 (6%) patients who seroconverted after the third dose (Figure 1): this was a 30-year-old male with RRMS (EDSS: 3.0, disease duration 4.4 years, and OCR treatment for 20 months at the time of third vaccination, time between second and third vaccination: 19 weeks). His lymphocyte count was 1.3 (normal value 0.8–4 G/L) at the timepoint of third vaccination and FACS revealed a B lymphocyte count of 132/µL (normal value: 80–616/µL). He increased his antibody titer from 3 AU/mL to 493 AU/mL 4.2 weeks after the third dose. The first antibody test took place 18 weeks after the second vaccination, whereas the second antibody test took place 4 weeks after the third vaccination.

## 4. Discussion

In this monocentric study, we evaluated the humoral immune response in pwMS treated with the CD20 depleting monoclonal antibodies OCR or RTX after a second and third dose of an mRNA-based SARS-CoV-2 vaccine. After the second dose, only two out of 49 patients (2.3%) had a positive IgG response against the Spike protein. Our Swiss data are at the lower end of previously reported data from Israel, Italy, and the United States [8,9,10]. There, seroconversion rates were 3.8%, 36%, and 36% respectively. These differences between the studies can probably be explained by different vaccination dates and different times of antibody testing, as age, sex distribution, and disability do not differ substantially. Currently, in patients who do not have a sufficient humoral response after a second vaccination and, thus, may not have adequate protection against COVID-19 infection, a third vaccination is recommended to obtain protection [24,25].

Consistently with the findings after the second immunization course, the success for seroconversion after a third vaccine dose was modest. Only one out of 16 patients (6.3%) in the cohort of previously lacking IgG responses to spike protein after two courses of mRNA vaccination developed a protective humoral immune response. This is surprising, as recent data from French kidney transplant patients were encouraging, showing a positive humoral response in 49% of patients who received the third homonologous vaccination with mRNA-1273 [26]. However, none of these patients had been treated with CD20 depleting therapies. Furthermore, it is unclear why our patient did not show seroconversion after the first and second vaccination, but after the third. B-cell counts at the time of the first and second vaccination are not available. As current data show that antibodies decrease over time, it is possible that he already developed a low humoral response after the second vaccination, which was no longer detectable with the first antibody test 18 weeks after the second vaccination [27]. In contrast, the second antibody test took place 4 weeks after the third vaccination. As there is a correlation between antibody titer against the Spike protein and protection against COVID-19 or a severe course, factors influencing the development of antibodies should be urgently investigated further [22]. There is also an ongoing debate on whether the use of a heterologous vaccine for the third immunization course would make sense if no IgG response was observed after two courses of the vaccination [28].

In our study, the maximum time between the last administration of the B cell-depleting medication and the third vaccination was 31.3 weeks (median 22.9). This comparatively shorter time frame may, in part, explain the limited seroconversion rates, as recent studies found a more effective humoral immune response after longer intervals of up to 144 weeks for RTX and 62.8 weeks for OCR treated patients vaccinated with mRNA vaccines [10,29]. Therefore, if clinically justifiable, extending the infusion interval between anti-CD20 antibody infusions and/or between the vaccine doses should be considered to increase the chances of successful vaccination against SARS-CoV-2 [30]. However, it is currently unclear how long the subsequent administration of CD 20 depleting therapies should be postponed, as a recently published study found no positive effect after an interval of up to 43 weeks in 60 RRMS patients vaccinated with mRNA vaccines [31]. There is emerging evidence in pwMS that the extent of vaccine-induced humoral responses correlates better with the extent of B-cell reconstitution at the time of vaccination than with the time window between the last anti-CD20 infusion and vaccination [32]. This observation is also corroborated by our study, even if seroconversion with the third vaccination was only observed in a single patient. Thus, vaccination based on the onset of B cell reconstitution, instead of adherence to a time window could be a way forward.

Limitations of our study include the small sample size, and the lack of data on T cell responses, given that the humoral immune response is complemented by cellular immunity to provide protective immune responses following vaccination. It has been shown that early and robust T-cell responses are present in mild/asymptomatic COVID-19 infection, even in the absence of antibodies [33]. Low antibody titers, therefore, cannot be equalized with lacking immunity. As a matter of fact, CD 4 and CD8 T-cell responses to SARS-CoV-2 after vaccination with BNT162b2 of OCR-treated pwMS were comparable to those of healthy individuals, even when antibody levels were lower or absent [34,35,36]. This suggests that vaccination of patients with B-cell deficiencies probably still provides some level of immunity against SARS-CoV-2, especially considering that T cells may also recognize newly emerging variants that have escaped neutralization by antibodies [37,38]. Moreover, there is the hypothesis that an attenuated development of S1-IgG on CD20 depleting antibody therapy may be counterbalanced by exaggerated T-cell responses [35]. The potential underlying immunological mechanism is a more extensive antigen-driven CD8 T cell activation without antibody-mediated antigen clearance. Further, disinhibition of CD8 T cell responses by CD20-depletion related lack of regulatory B cells can be envisioned [35].

Answering these questions is of great importance to pwMS, given that CD20 depleting therapies are a mainstay of disease-modifying treatment and prevention of infections are of major importance. Efforts need also to be directed towards the questions of why individuals treated with CD20 depleting antibodies are at higher risk for unfavorable outcome of COVID-19. Furthermore, studies are needed to evaluate whether the third vaccination with another preparation leads to better results regarding antibody response and the role of T cells in the protection against SARS-CoV-2 infection, and if measurement of CD19 cells as point-of-care measure might allow to better define the best time window for effective vaccination in patients treated with CD20 depleting therapies.

## 5. Conclusions

The development of a humoral immune response remains rare in pwMS on anti-CD20 therapy even after third dose of the homologous SARS-CoV-2 mRNA vaccine. It remains to be determined whether T-cell responses can compensate the lacking seroconversion and provide sufficient protection against CoV-2 infections.

## Figures and Tables

**Table 1:** Demographics, disease characteristics, and lymphocyte counts in people with MS receiving the second and third SARS-CoV-2 mRNA vaccination.

	Patients after 2nd COVID-19 Vaccination	Patients after 3rd COVID-19 Vaccination
Patients	n = 49 (f = 28 (68%))	n = 16 (f = 9 (56%))
	RRMS n = 34	RRMS n = 9
MS course	SPMS n = 5	SPMS n = 2
	PPMS n = 10	PPMS n = 5
Mean age (SD)	47.4 years (12.4)	51.0 years (12.3)
Mean disease duration (SD)	12.4 years (8.1)	13.56 years (8.2)
Median EDSS (range)	3.0 (0–7.5)	3.0 (1.0–7.5)
Median time between last vaccination and antibody testing (range)	18.9 weeks (4.3–28.4) *	4.4 weeks (3.7–7.3) **
Median lymphocyte count at time of antibody testing (range) ***	1.4 (0.7–2.8) ^(1)^	1.5 (0.8–2.7) ^(2)^

Table legend: f = female; SD = standard deviation; RRMS = relapsing–remitting MS; SPMS = secondary-progressive MS; PPMS = primary-progressive MS; * median time between 2nd vaccination and Spike-protein IgG testing; ** median time between 3rd vaccination and Spike-protein IgG testing; *** normal values: 0.8–4 G/L; ^(1)^ n = 44; ^(2)^ n = 12.

## Data Availability

The data that support the findings of this study are available on reasonable request from the corresponding author.
